# Knowledge, Attitude and Perception of Risk and Preventive Behaviors toward Premarital Sexual Practice among In-School Adolescents

**DOI:** 10.3390/ejihpe10010036

**Published:** 2020-03-01

**Authors:** Shamsudeen YAU, Pramote Wongsawat, Archin Songthap

**Affiliations:** Department of Community Health, Faculty of Public Health, Naresuan University, Phitsanulok 65000, Thailand

**Keywords:** adolescent, knowledge, attitude, perception, premarital sexual practice, risk and preventive behaviors

## Abstract

Premarital Sexual Practice (PSP) among adolescents usually involves sexually risky behaviors, such as multiple sexual partners and inconsistent or non-condom use. These behaviors, in combination with other underlining factors, undermine the overall outcomes of Adolescent Sexual and Reproductive Health (ASRH). To assess the adolescents’ knowledge, attitudes and perception of risk and preventive behaviors towards PSP, a school-based analytical cross-sectional study was conducted among a sample of 423 students aged 15 through 19 years. A well-validated anonymous self-administered questionnaire was used for collecting the data, which were analyzed using mean (SD), frequency (%), t-test, ANOVA and multiple regression methods. Participants’ knowledge of risk and preventive behaviors was average, as only 53% of knowledge items were correctly answered. Being a female, of high-income status, in the second study year, perceived susceptibility and perceived severity were significant determinants of knowledge. All measures of perception except perceived self-efficacy were positive determinants of attitude. Being female, in the third study year and of high-income status were determinants of perception as measured by perceived self-efficacy. Therefore, our results suggest that tailored educational programs, with special emphasis on financially disadvantaged male adolescents, are needed to effectively increase adolescents’ knowledge, attitude and perception of risk and protective behaviors towards PSP.

## 1. Introduction

Premarital Sexual Practice (PSP) is a common sexual experimentation among adolescents [1,2], which usually involves behaviors such as multiple sexual partners [3,4], inconsistent or non-condom use [5] and consumption of pornography [6,7]. Other dangerous behaviors are illicit drug use [8] and the use of alcohol [4,7]. Furthermore, sexual intercourse during the teenage period increases the tendencies for high-risk sexual behaviors such as prostitution and being promiscuous in adulthood [9]. These sexually risky behaviors, in combination with other underlining factors, undermine the overall outcomes of Adolescent Sexual and Reproductive Health (ASRH) [10].

Additionally, early sexual activities, which are often unprotected, have been heavily linked to consequences such as unwanted pregnancies [10]. Annually, an estimated 21 million girls aged 15 to 19 years get pregnant in developing nations, and nearly half of these pregnancies are unintended [11,12]. Of these, between 12–16 million give birth, accounting for about 11% of global births. Unfortunately, developing countries, most of which are in Africa and Asia, account for 95% of the global births by adolescents [13]. These pregnancies are often associated with ante-, intra- and/or post-partum complications [14,15,16], and infection tendencies are higher among younger women. Seemingly, early childbearing has been linked to impeding adolescents’ psychological, physical, economic and emotional development [17].

For decades, PSP has been a global health concern, with variations in perception and acceptance between cultures across countries [18,19,20]. In Thailand, PSP has, in recent times, been trending, receiving widespread popularity and acceptance among Thai teenagers across the country [21]. This has been reflected in the consistent decline of age at first sexual debut in recent years [22]. Previously, the first sexual encounter among this age group usually occurred between the ages of 14.5–16.7 [23]. However, according to the Ministry of Public Health (MoPH), the current national average age of sexual initiation is 13 years among adolescent girls and is even lower for boys.

Research regarding adolescent perception and practices as they relate to PSP has previously documented various sexually risky behaviors among Thai adolescents but rarely among vocational schools students. Although inconsistent condom use, multiple sexual partners and higher risks of earlier onset of sexual activities are prevalent among Thai adolescents [24,25], the risks are even higher among vocational school adolescents. Against 1 in 3 for boys and 1 in 7 for girls of general school adolescents, nearly 2 in 3 vocational school male and female students have had premarital sex; they are also significantly more likely to have multiple sexual partners and not to use condoms consistently [25]. Therefore, with this increasing acceptance of PSP among adolescents, it is imperative to assess and understand their knowledge, attitude and perception toward PSP. The research question for the study was: what are the levels of knowledge, attitude and perception of risk and preventive behaviors towards PSP among vocational school adolescents? The outcomes of the study could inform future intervention strategies specifically designed to mitigate the negative impacts of PSP among Thai adolescents.

## 2. Materials and Methods 

### 2.1. Study Design, Settings and Participants

This was a school-based analytical cross-sectional study conducted in the Mueang District (Municipality District) of Phitsanulok Province between April and May 2019. Initially, a total of six (6) vocational schools were identified and clustered (those within vs. outside the Municipality District of the Province). Both clusters ended up with three schools each. The schools within the boundaries of the Municipality District, the province’s biggest district, were purposively considered for this research. A sample size of 423 was determined based on the finite population proportion formula. A multistage random sampling method was used to select the participants. The 423 samples were drawn from the three participating schools proportionate to the population sizes of their students. Each of the selected schools was then stratified according to the academic year of study (year I, year II and year III). From each academic year, a sample from both sexes was drawn proportionate to the gender ratio. The inclusion criterion was that the students must be unmarried and between 15 through 19 years. The research was conducted in full accordance with the Declaration of Helsinki, and approved by the Institutional Review Board (IRB) of Naresuan University, with certificate of approval (CoA) number 0058/2019.

### 2.2. Research Tool

The study tool was developed using the Health Belief Model (HBM) as the theoretical framework [26]. The HBM constructs relevant to our research questions were adopted to develop the questionnaire, which was initially developed in the English language but administered in the respondent Language, Thai. Back translation was used to ensure that the meaning and substance of the items were not significantly altered. The questionnaire consisted of four parts, namely: (1) general characteristics, (2) knowledge, (3) attitude and (4) perception. The questionnaire items were formed based upon an intensive review of previous research. Multiple choice questions (four options; one correct and three wrong answers) were used for the 17 items for knowledge. A correct answer was assigned the score of ‘1’ while any of the three wrong answers were assigned ‘0’. Thus, the possible scores ranged between 0–17. The overall mean score was used for the final analysis. Additionally, the items for attitude and perception were measured using the 5-point Likert scale. The scale for attitude, perceived susceptibility and perceived severity was thus: 5: Strongly Agree; 4: Agree; 3: Neutral; 2: Disagree; and 1: Strongly Disagree. Perceived self-efficacy was measured as 5: Can do it surely; 4: Can do it; 3: Not sure; 2: Cannot do it; and 1: Cannot do it surely. Negative questions were transformed (‘strongly disagree’ was coded 5 while ‘strongly agree’ was coded 1). The possible scores ranged between 10–50 for attitude (10 items); 10–50 for perceived susceptibility (10 items); 6–30 for perceived severity (6 items); and 8–40 for perceived self-efficacy (8 items). The overall mean scores for both attitude and perception were also used for the final analysis. The higher the participants’ attitude score, the more conservative was their attitude toward PSP. Likewise, the higher the perception score, the more understanding they were of the consequences of PSP.

Prior to the data collection exercise, three experts assessed and evaluated the content validity of the tool. Only items with an Item Objective Congruence (IOC) index of > 0.5 were retained. The reliability of the tool was also examined by pilot-testing it on 47 students (10% of the study sample) who were of comparable characteristics to the participants of the main study. The reliability test yielded a Chronbach alpha of 0.706 for knowledge, 0.870 for attitude and 0.936 for perception.

### 2.3. Data Collection

The data collection was carried out in the three selected schools, where the questionnaire was self-administered to the students. While responding to the questionnaire, participants were urged not to engage in discussions with their friends to ensure accuracy of information free of external influence. Students were advised to sit far apart from one another to maximize the privacy of information. Additionally, to minimize recall bias, responding to the questionnaire was self-paced to enhance accurate recollection of past events.

### 2.4. Data Analyses 

The data were analyzed using a statistical software program (SPSS^®^, version 20.0). Descriptive statistics were utilized to describe the participants’ socio-demographic variables. Knowledge was analyzed and presented by means and frequencies item-by-item. Then, the overall mean knowledge score was computed and presented by mean (SD) and percentages. Independent samples t-test and ANOVA were used to test the overall mean knowledge score against other socio-demographic variables for potential associations in a univariate analysis. Assessment of attitude and perception was carried out by averaging participants’ responses of each item on the 5-point scale and presenting it as mean (SD). The overall mean scores for both attitude and perception were also calculated and presented by mean (SD). Independent samples t-test and ANOVA were again used to examine the possible relationships between attitude and perception and socio-demographic variables in a univariate analysis. Finally, variables that were significantly associated with the outcomes and not considerably correlated were included in a multivariate analysis (linear regression) to determine factors associated with participants’ knowledge, attitude and perception toward PSP. All analyses, except otherwise indicated, were performed at a 5% level of statistical significance with a 95% level of confidence.

## 3. Results

### 3.1. Socio-Demographic Characteristics

The participants were distributed into two equal halves; 50.1% male and 49.9% female, with the mean age of 16.79 ± 1.07 (male: 16.84 ± 1.10; female: 16.74 ± 1.05). An overwhelming number of them (98%) were Buddhists and nearly two-thirds were first year students and one-quarter were in the third year (Table not shown). The majority (47.3%) were studying in a science cluster (science courses), 30.7% were studying in a commercial cluster and 22% were studying in an arts cluster. The average monthly income was 3389.95 ± 2369.12 Thai baht (male: baht 3481.99 ± 2798.99; female: baht 3297.44 ± 1841.58) and more than half (57.3%) received income from both parents. Furthermore, the residential areas of the participants were sub-district administrative organization area (41.4%), sub-district municipality area (28.9%) and municipality area (29.7%). 

### 3.2. Knowledge of Risk and Preventive Behaviours

The overall mean knowledge score was 9.06 ± 3.63 out of a total of 17 (Table 1). The proportions of participants who correctly answered the knowledge items and the mean score of each item are presented. On average, only 53% of the knowledge items were correctly answered by the participants (49% males vs. 57.5% females; P < 0.001; Table not shown). Generally, adolescents were more likely to score above average in questions related to the effectiveness of condoms in HIV/STDs prevention (0.99 ± 0.101), the health impact of HIV (0.79 ± 0.408), preventability of STDs (0.73 ± 0.442), pregnancy risk behavior (0.62 ± 0.487), risky sexual behaviors (0.61 ± 0.489), HIV risk behavior (0.60 ± 0.491), consequences of teenage pregnancy (0.55 ± 0.498) and HIV treatment (0.54 ± 0.499) and testing (0.54 ± 0.499). They performed the worst in questions related to the mode of transmission of HIV (0.22 ± 0.415), skilled condom use (0.24 ± 0.426), advantages of condoms over pills (0.35 ± 0.476) and pregnancy preventive behavior (0.39 ± 0.487). 

### 3.3. Attitude toward PSP

The overall attitude mean score was 3.41 ± 0.60 out of 5 (Table 2). For the majority of the items, participants’ scores were higher than three, indicating a conservative attitude towards PSP. Reasons for these high levels of attitude were the adolescents’ concern about hurting their parents, fear of pregnancy and infection and fear of losing one’s virginity. On the other hand, a more liberal attitude was seen among participants who believed that having premarital sex strengthens bonding between lovers and those whose peers were engaged in sexual activities. 

### 3.4. Perception

#### 3.4.1. Perceived Susceptibility

As shown in Table 3, the overall mean score of perceived susceptibility was 3.65 ± 0.56 (Table 3). For the majority of the items, participants’ perceived susceptibility was significantly high. Adolescents perceived that situations that could potentially lead to PSP were being in isolation with the opposite sex, dressing in a sexually offensive manner, having a boy/girlfriend, alcohol drinking, watching pornography and drug use. Participant also understood that engaging in PSP predisposes one to risks of teenage pregnancy and HIV/STIs. 

#### 3.4.2. Perceived Severity

The overall mean score of perceived severity was 3.38 ± 0.67 (Table 4). Participants strongly perceived the severity of unsafe abortion, teenage pregnancy and the financial consequence of PSP. However, they weakly perceived the severity of pregnancy complications in teenagers, and the health impact of HIV and STIs.

#### 3.4.3. Perceived Self-Efficacy 

The overall mean score of perceived self-efficacy was 3.82 ± 0.74 (Table 5). Participants showed that they were capable of: declining boy/girlfriend’s invitation to nightclubs; as well as rebuking obscenity from their boy/girlfriends and forbearing boy/girlfriends’ invitation to visit their home alone. They also demonstrated that they were able to: resist touch, hugs or kisses from boy/girlfriend; refuse sexual conversations with boy/girlfriend; and avoid an opportunity to be alone with boy/girlfriends. However, they scored slightly lower in the ability to have control over their sexual desire and the confidence to deny intercourse to their boy/girlfriends.

### 3.5. Factors Associated with Knowledge, Attitude and Perception toward PSP

#### 3.5.1. Knowledge of Risk and Preventive Behaviors

Table 6 and Table 7 demonstrate the univariate relationships between knowledge, attitude and perception with certain socio-demographic variables. In the multivariate analysis, knowledge was associated with gender, study year, income and perception (Table 8). Accordingly, the mean score of knowledge increased by 1.43 for a unit change in gender (from male to female) (P = 0.001). This means that females were significantly more knowledgeable regarding risk and preventive behaviors toward PSP. Conversely, knowledge decreased by 1.88 for a unit change in study year (from 1st year to 2nd year) (P < 0.001). Additionally, a unit change in income (from low to high) was associated with a 1.08 increase in the mean score of knowledge (P = 0.034). Similarly, while a unit change in perceived susceptibility was associated with a 0.81 increase in the mean score of knowledge (P = 0.044), a unit change in perceived severity appeared to decrease the mean score of knowledge by 1.53 (P < 0.001).

#### 3.5.2. Attitude toward SPS

The attitude was significantly associated with perception (Table 8). For a unit change in perceived susceptibility and perceived severity, the mean score of attitude increased by 0.36 and 0.30, respectively (P < 0.001). This means that participants with conservative attitudes toward PSP had higher perceived susceptibility to and perceived severity of PSP.

#### 3.5.3. Perception

The mean score of perceived susceptibility increased by 0.01 (P = 0.044), 0.29 (P < 0.001), 0.25 (P < 0.001) and 0.20 (P < 0.001) for a unit change in knowledge, attitude, perceived severity and perceived self-efficacy, respectively (Table 9). This implies that respondents with a high perceived susceptibility to PSP were more likely to have high knowledge, attitude, perceived severity and perceived self-efficacy. The mean score of perceived severity, on the one hand, decreased by 0.04 (P < 0.001) for a unit change in knowledge. On the other hand, it increased by 0.36 (P < 0.001) and 0.37 (P < 0.001) for a unit change in attitude and perceived susceptibility, respectively. This translates that the relationship between perceived severity and knowledge was inverse, but it was directly related to attitude and perceived susceptibility. Finally, the mean score of perceived self-efficacy increased by 0.29 (P < 0.001) and 0.46 (P < 0.001), respectively, for a unit change in gender (from male to female) and perceived susceptibility. Contrastingly, a unit change in study year (from 1^st^ year to 3^rd^ year) and income (from low to high), showed a decrease in the mean score of self-efficacy by 0.24 (P = 0.034) and 0.20 (P = 0.049), respectively. 

## 4. Discussion

### 4.1. Knowledge of Risk and Preventive Behaviours

The present study examined the participants’ knowledge, attitude and perception of risk and preventive behaviors towards PSP. Overall, adolescents’ knowledge of risk and preventive behaviors toward PSP was generally average (53%). This low level of knowledge may reflect the widespread risky sexual behaviors, such as promiscuity, inconsistent condom use and deliberate refusal to test for HIV, among sexually experienced Thai youths [27,28]. Arguably, adequate knowledge of risk and preventive behaviors toward PSP is essential to the containment of the undesirable consequences of PSP among teenagers. Therefore, adolescents must be adequately equipped with quality knowledge of risk and protective behaviors toward premarital sex, as well as its consequences, to enable them to make informed decisions that could directly or indirectly affect their overall reproductive health. 

However, despite this knowledge inadequacy among the respondents, we found that an overwhelming number of them (99%) were correctly enlightened about condom effectiveness in the prevention of HIV/STDs. Further, a significant number (73%) correctly thought that all STDs are entirely preventable, of which HIV/AIDS is the most life-threatening. This is reasonably accurate because although other STIs, such as Chlamydia and Human Papilloma Virus (HPV), pose various degrees of sexual health problems including vulnerability to HIV infections, fatalities from these STIs are less common. In terms of the risks of PSP, adolescents admittedly recognized that engaging with peers in activities such as alcohol drinking, dining out and being alone in secluded places influence PSP. It is likely that adolescents may tend to be more reckless when they are drunk or secluded and, probably because they are rationally less composed, they engage in risky sexual activities. This aligns with previously reported evidence of the impact of peer influence and alcohol drinking on premarital sex among adolescents [4,6,7]. Conversely, adolescents were not adequately informed about HIV treatments as only half of them (54%) accurately understood that viral suppression is achievable with the right treatment regimen and appropriate counseling from a trained medical practitioner. This was supported by a randomized controlled trial which has shown that timely medication with the right regimen, such as the antiretroviral therapy (ART), has the potential to avert over 93% of partner transmission as a result of viral suppression [29]. 

Most importantly, the vast majority of adolescents wrongly conceived that one could be infected with HIV by sleeping in the same room with an infected person. This concords with the growing body of evidence regarding the misconception of HIV transmission among adolescents from various regions of the world [30,31,32]. Similarly, barely a third of adolescents truly understood that condoms are more advantageous than oral contraceptives. Factually, oral contraceptives could prevent pregnancy but not infections, while condoms prevent both when used correctly, but with a little less precision. Participants poorly understood the risks and consequences of teenage pregnancy, as barely half of them correctly acknowledged that unsafe abortion could lead to infections, possible blood loss and uterine rupture. Practically, all of those could be associated with unsafe abortion. Empirical evidence from the findings of a qualitative Iranian study reported that, following unsafe abortion, the risks of severe blood loss, uterine rupture and infection are high [33]. Further evidence from an Indian study has claimed unsafe abortion as a possible cause of maternal mortality [34]. This lack of knowledge highlights the need for concerted efforts between educators and health authorities to educate adolescents about risky sexual behavior, different contraceptive options and their effectiveness in preventing both pregnancy and infections.

### 4.2. Attitude toward PSP

Participants demonstrated a high level of conservative attitude toward PSP for fear of pregnancy and infection and because they were particularly concerned that it will be hurtful to their parents when they find out. This has been evidenced in a previous study where sexually inexperienced adolescents admitted to delaying sexual intercourse because they are particularly concerned about the feelings of their parents [35]. Perhaps this level of concern may have an indirect bearing on the expectations of sexual inactivity that Thai society places on female children in order not to ruin the family reputation. Preceding evidence has shown that sexually experienced girls lose societal dignity and are gossiped about, which is shameful and embarrassing to the entire family [21]. Additionally, due to a conscious desire to preserve their virginity, adolescents, especially females, displayed a high conservative attitude toward PSP. This agrees with a study among young Iranian adolescents, where females were more conservative of their virginity than males [36]. In the Thai context, however, it could perhaps be attributed to the fact that Thai society seems to be more judgmental toward girls’ sexual life than boys’. Unlike the boys, a girl’s virginity is considered her valuable asset which she is expected to preserve until marriage [21]. In summary, males appeared to be more permissive toward PSP than females, which corroborates preceding evidence from different parts of the globe [36,37].

### 4.3. Perception

Perceived susceptibility to premarital sex was appreciably high. Adolescents perceived they were susceptible to PSP when they are in seclusion with the opposite sex, having a boyfriend, or consuming pornography. This is particularly reasonable because high sexual arousal is likely when watching pornography, which may influence engagement in premarital sex. A growing body of evidence has consistently linked the consumption of erotic materials [6,7,38,39] and having a boyfriend [4] to PSP among adolescents. With regard to the perceived severity of PSP and its consequences, adolescents seemed aware of the impact of teenage pregnancy and HIV/STIs; although a close examination showed that males outperformed females in perceived severity of STIs. This was not anticipated given that the prevalence of STI among 15–19 years old adolescents is much higher in females than males [40,41]. Therefore, it could be correctly expected that females may have greater experience of the severity of these infections than males. Perceived self-efficacy was higher than the other two measures of perception. Most adolescents demonstrated the ability to have absolute control over their sexual desire, even in the presence of an opportunity to satisfy it, and were resilient to refuse sexual advances from the opposite sex. This was consistent with previous findings which suggested that high self-efficacy delays sexual initiation and promotes safe sex practices like the use of condoms [42,43,44]. 

### 4.4. Factors Associated with Knowledge, Attitude and Perception

As seen in the univariate analysis of knowledge, the gender difference was well elaborated in the overall knowledge score, which significantly favored the female gender. However, this contradicts previous studies, which suggested that knowledge of HIV and other sexual behaviors was higher among male adolescents [45,46]. The findings of this study could be due to the fact that Thai parents may preferentially place more emphasis on girls’ sexual education than boys’, because evidence of deep-rooted double standards regarding sexual behaviors as applied to boys on the one hand and girls on the other has been documented [21]. A significantly higher knowledge was observed among the first year than the second year students. This disagrees with preceding studies that attributed higher sexual knowledge to upper study years [47,48]. Additionally, it was not unexpected that participants in the higher income group were considerably more informed than participants in the low-income group, because prior studies have associated wealth index with HIV knowledge [49], perhaps because access to information, whether through electronic or print media, could be a lot easier and more affordable for adolescents that are financially well-off. Furthermore, although knowledge appeared to positively correlate with perceived susceptibility, the correlation with perceived severity, however, was negative. On the other hand, beside perceived self-efficacy, attitude was positively correlated with all measures of perception. This could plausibly be true because the high perception of the severity of consequences of PSP—such as HIV/STDs infection, teenage pregnancy, etc.—could translate into a higher perception of the susceptibility to these consequences, which may, in turn, neutralize intentions to engage in PSP.

Furthermore, the higher self-efficacy observed among females showed that they were significantly more able than males to decline sexual advances. Although this could not be comparatively ascertained due to lack of prior evidence, it is reasonably understandable given that the females in our study were more informed regarding risk and preventive behaviors, which might have afforded them this advantage over their male counterparts. On the reverse side, self-efficacy was lower for students belonging to the third year of study and high-income group. The poor self-efficacy among the third year students—who were also older—might reflect the consistently risky sexual activities associated with late adolescence [50]. Furthermore, as opposed to the low-income adolescents, high-income earners might not be as much concerned about the aftermath of PSP, since, in spite of the legal implications of abortion in Thailand, it is still relatively easily accessible by the financially privileged tier of Thais [51].

## 5. Conclusions

Although adolescents’ knowledge of risk and protective behaviors was generally insufficient, perceived self-efficacy was reasonably sufficient, with females outperforming males. Attitude and other measures of perception (perceived severity and perceived susceptibility) were notably uninfluenced by most of the explanatory variables. Therefore, our results suggest that prevention programs that incorporate sexual education should tailor their materials to focus more on improving the risks knowledge and the preventive skills of adolescents, with special emphasis on male adolescents and particularly those who are financially disadvantaged. This could be an effective way to increase adolescents’ knowledge, attitudes and perceptions of risk and protective behaviors to enable them to make healthy decisions regarding PSP.

## 6. Strengths and Weaknesses

As part of its strength, the study has provided evidence of factors influencing knowledge, attitude and perception of vocational school adolescents towards PSP, which was apparently lacking even among the general Thai population. It further suggests that this is an understudied area, and that future studies should explore in-depth more explanatory factors other than the demographics so as to better understand influencers of and barriers to adequate knowledge, attitude and perception towards PSP. One of the limitations of the study, however, was that due to the lack of relevant references from the Thai settings, conclusions were drawn from studies in populations that could be dissimilar to the Thai population. It should also be noted that our study used retrospective questioning, which could potentially pose a problem of recall bias. Lastly, a full instrument validation trial was not performed, so it was not ascertained if the translation and norming were accurate.

## Figures and Tables

**Table 1:** Correct knowledge of risk and preventive behaviors toward premarital sexual practice across gender (**n** = 391).

Knowledge Items	n (%)	$\bar{X}$ (SD)
1. HIV is the most dangerous STI to health.	309 (79.0)	0.79 (0.408)
2. Correct medication can decrease viral load.	211 (54.0)	0.54 (0.499)
3. All STDs are preventable.	287 (73.4)	0.73 (0.442)
4. HIV decreases immune functions and increases other types of infections.	173 (44.2)	0.44 (0.497)
5. HIV is not transmittable by sleeping in the same room with an infected person.	86 (22.0)	0.22 (0.415)
6. HIV risky behaviors.	234 (59.8)	0.60 (0.491)
7. When to get tested for HIV/STIs or pregnancy.	210 (53.7)	0.54 (0.499)
8. Consequences of teenage pregnancy.	214 (54.7)	0.55 (0.498)
9. Teenage pregnancy risky behavior.	241 (61.6)	0.62 (0.487)
10. Consequences of unsafe abortion.	191 (48.8)	0.49 (0.501)
11. Condom effectiveness in HIV/STDs prevention.	387 (99.0)	0.99 (0.101)
12. Advantages of condom over oral contraceptives.	135 (34.5)	0.35 (0.476)
13. Skilled condom use.	93 (23.8)	0.24 (0.426)
14. Effective use of emergency pills.	192 (49.1)	0.49 (0.501)
15. Teen pregnancy preventive behaviors.	151 (38.6)	0.39 (0.487)
16. Risky sexual behaviors.	237 (60.6)	0.61 (0.489)
17. Safe sex practice.	190 (48.6)	0.49 (0.500)
Total mean score (SD)	9.06 ± 3.63	
Total % of correct answers	53%	

**Table 2:** Mean score of attitude towards Premarital Sexual Practice (PSP) (n = 391).

Attitude Items	$\bar{X}$ (SD)
1. It is normal for teenagers to have sex at school age.	3.32 (1.05)
2. Having sex at school age can strengthen lovers’ bond.	2.83 (1.14)
3. Having sex at school age can be done because my peers do it.	2.83 (1.11)
4. Having sex at school age can be done because it is common.	3.26 (1.05)
5. Having sex at school age is personal and does not affect anyone.	3.18 (1.02)
6. Having sex at school age is wrong because it is the age a student should study for the future.	3.66 (1.05)
7. Having sex at school age is not a suitable behavior.	3.64 (0.98)
8. Having sex at school age is wrong, it might lead to pregnancy and/or infection.	3.80 (0.99)
9. Having sex at school age is bad because it leads to loss of virginity.	3.70 (0.98)
10. Having sex at school age is wrong because it hurts parents.	3.83 (1.00)
Overall Mean (SD)	3.41 (0.60)

**Table 3:** Participants perception: mean score of perceived susceptibility to PSP (n = 391).

Perceived Susceptibility Items	$\bar{X}$ (SD)
1. Having a boy/girlfriend at school age is a risk for premarital sex.	3.75 (0.82)
2. Seclusion with the opposite sex can lead to premarital sex.	3.82 (0.82)
3. Premarital sex at school age increases the risk of HIV/STIs.	3.68 (0.80)
4. Premarital sex at school age increases the risk of pregnancy.	3.77 (0.89)
5. Sex education can reduce the risk of premarital sex at school age.	3.78 (0.84)
6. Pregnancy has no consequence for unmarried adolescents.	2.99 (1.11)
7. Sexually provocative dress can induce premarital sex at school age.	3.76 (0.88)
8. Drinking alcohol can lead to premarital sex at school age.	3.71 (0.92)
9. Drug abuse/addiction can lead to premarital sex at school age.	3.61 (0.92)
10. Watching pornography can result in premarital sex at school age.	3.62 (0.96)
Overall mean (SD)	3.65 (0.56)

**Table 4:** Participants perception: mean score of perceived severity of PSP (n = 391).

Perceived Severity	$\bar{X}$ (SD)
1. Teenage pregnancy can cause adverse health impacts.	3.13 (1.10)
2. Unsafe abortion can result in many health-damaging consequences.	3.81 (0.96)
3. HIV infection is injurious to health and quality of life.	3.13 (1.20)
4. STIs are damaging to health and quality of life.	3.02 (1.14)
5. Teenage pregnancy can affect adolescents’ life planning choices.	3.69 (0.90)
6. PSP can affect the economic status of teenagers and their families.	3.51 (0.94)
Overall Mean (SD)	3.38 (0.67)

**Table 5:** Participants perception: perceived self-efficacy regarding PSP (n = 391).

Perceived Self-Efficacy	$\bar{X}$ (SD)
1. Rebuke obscene talk from my girl/boyfriend.	3.93 (0.88)
2. Refuse sexual conversations with my girl/boyfriend.	3.81 (0.88)
3. Disregard my girl/boyfriend’s invitation to their house.	3.87 (0.95)
4. Resist touches, hugs or kisses from girl/boyfriend.	3.85 (0.96)
5. Reject girl/boyfriend’s invitation to nightlife venues.	3.95 (0.93)
6. Prevent being alone with girl/boyfriend.	3.80 (0.93)
7. Restrain my sexual desires.	3.75 (0.97)
8. Deny my girl/boyfriend’s request to have sex.	3.59 (1.07)
Overall Mean (SD)	3.82 (0.74)

**Table 6:** Mean scores of knowledge and attitude by socio-demographic variables (n = 391).

Variables	n (%)	Knowledge		Attitude	
		Mean (SD)	P-Value	Mean (SD)	P-Value
**Gender**					
Male	196 (50.1)	8.33 (3.59)	< 0.001 *	3.44 (0.60)	0.239
Female	195 (49.9)	9.78 (3.54)		3.37 (0.59)	
**Age (years)**					
15	32 (8.2)	9.13 (3.34)	0.045 ^#^	3.20 (0.65)	0.013 ^#^
16	151 (38.6)	9.05 (3.81)		3.46 (0.58)	
17	102 (26.1)	8.28 (3.68)		3.29 (0.66)	
18	79 (20.2)	9.59 (3.38)		3.54 (0.51)	
19	27 (6.9)	10.33 (3.0)		3.38 (0.49)	
**Study Year**					
First	235 (60.1)	9.09 (3.66)	< 0.001 ^#^	3.37 (0.59)	0.189
Second	61 (15.6)	7.59 (3.53)		3.38 (0.72)	
Third	95 (24.3)	9.92 (3.35)		3.50 (0.54)	
**Monthly Income (Baht)**					
Low	74 (18.9)	8.62 (3.37)	0.008 ^#^	3.42 (0.56)	0.845
Middle	199 (50.9)	8.70 (3.64)		3.42 (0.66)	
High	118 (30.2)	9.92 (3.66)		3.38 (0.53)	

Notes: α < 0.05, * Independent samples t-test, ^#^ ANOVA.

**Table 7:** Mean scores of perception by socio-demographic variables (n = 391).

Variables	n (%)	PERCEPTION					
		Perceived Susceptibility		Perceived Severity		Perceived Self-Efficacy	
		Mean (SD)	P-Value	Mean (SD)	P-Value	Mean (SD)	P-Value
**Gender**							
Male	196 (50.1)	3.67 (0.60)		3.46 (0.62)		3.70 (0.73)	
Female	195 (49.9)	3.64 (0.52)	0.599	3.29 (0.70)	0.010 *	3.94 (0.73)	0.001 *
**Age (years)**							
15	32 (8.2)	3.49 (0.54)		3.24 (0.64)		3.69 (0.84)	
16	151 (38.6)	3.67 (0.53)		3.41 (0.68)		3.91 (0.68)	
17	102 (26.1)	3.56 (0.67)		3.32 (0.67)		3.72 (0.79)	
18	79 (20.2)	3.73 (0.49)		3.45 (0.65)		3.85 (0.68)	
19	27 (6.9)	3.83 (0.44)	0.038 ^#^	3.38 (0.64)	0.484	3.75 (0.83)	0.254
**Religion**							
Buddhist	383 (98.0)	3.64 (0.56)		3.37 (0.67)		3.81 (0.74)	
Christian	08 (02.0)	4.04 (0.50)	0.049 *	3.79 (0.74)	0.078	4.27 (0.65)	0.083
**Study Year**							
First	235 (60.1)	3.62 (0.53)		3.37 (0.67)		3.85 (0.69)	
Second	61 (15.6)	3.60 (0.68)		3.39 (0.75)		3.84 (0.77)	
Third	95 (24.3)	3.76 (0.53)	0.082	3.39 (0.62)	0.960	3.72 (0.82)	0.299
**Study Major**							
Arts cluster	86 (22.0)	3.65 (0.55)		3.43 (0.69)		3.89 (0.72)	
Science cluster	185 (47.3)	3.64 (0.55)		3.37 (0.67)		3.70 (0.75)	
Commercial cluster	120 (30.7)	3.66 (0.56)	0.969	3.56 (0.65)	0.689	3.95 (0.71)	0.008 ^#^
**Monthly Income**							
Low	74 (18.9)	3.69 (0.46)		3.35 (0.60)		3.93 (0.71)	
Middle	199 (50.9)	3.63 (0.58)		3.41 (0.69)		3.84 (0.75)	
High	118 (30.2)	3.67 (0.58)	0.666	3.34 (0.67)	0.616	3.71 (0.73)	0.100

Notes: α < 0.05, * Independent samples t-test, ^#^ ANOVA.

**Table 8:** Factors associated with knowledge and attitude of adolescents toward PSP (n = 391).

Variables	Knowledge			Attitude		
	B	SE	P-Value	B	SE	P-Value
**Age (years)**	0.132	0.219	0.548	−0.029	0.031	0.361
**Gender**						
Male (ref)	0			0		
Female	1.428	0.352	0.001 *	−0.014	0.051	0.779
**Study Year**						
First (ref)	0			0		
Second	−1.876	0.516	< 0.001 *	0.037	0.075	0.624
Third	0.506	0.569	0.374	0.136	0.081	0.094
**Monthly Income (Baht)**						
Low (ref)	0			0		
Middle	0.071	0.460	0.878	−0.003	0.066	0.960
High	1.076	0.506	0.034 *	−0.023	0.073	0.750
**Knowledge**	-	-	-	0.000	0.007	0.962
**Attitude**	0.017	0.360	0.962	-	-	-
**Perceived Susceptibility**	0.807	0.399	0.044 *	0.358	0.054	< 0.001 *
**Perceived Severity**	−1.526	0.318	< 0.001 *	0.301	0.044	< 0.001 *
**Perceived Self-Efficacy**	−0.345	0.263	0.190	0.067	0.038	0.074

Notes: * α < 0.05,

**Table 9:** Factors associated with adolescents’ perception of PSP (n = 391).

Variables	Perception								
	Perceived Susceptibility			Perceived Severity			Perceived Self-Efficacy		
	B	SE	P-Value	B	SE	P-Value	B	SE	P-Value
**Age (years)**	0.010	0.028	0.733	0.025	0.034	0.460	0.034	0.043	0.381
**Gender**									
Male (ref)	0			0			0		
Female	−0.025	0.046	0.594	−0.097	0.056	0.086	0.286	0.069	< 0.001 *
**Study Year**									
First (ref)	0			0			0		
Second	0.009	0.067	0.894	−0.060	0.083	0.468	−0.084	0.103	0.413
Third	0.101	0.073	0.169	−0.108	0.089	0.229	−0.235	0.111	0.034 *
**Monthly Income (Baht)**									
Low (ref)	0			0			0		
Middle	−0.073	0.059	0.217	0.106	0.072	0.144	−0.056	0.090	0.531
High	0.001	0.065	0.990	0.089	0.080	0.266	−0.195	0.099	0.049 *
**Knowledge**	0.013	0.007	0.044 *	−0.038	0.008	< 0.001 *	−0.013	0.010	0.190
**Attitude**	0.289	0.044	< 0.001 *	0.363	0.053	< 0.001 *	0.126	0.070	0.074
**Perceived Susceptibility**	-	-	-	0.371	0.060	< 0.001 *	0.463	0.075	< 0.001 *
**Perceived Severity**	0.248	0.040	< 0.001 *	-	-	-	0.057	0.064	0.375
**Perceived Self-Efficacy**	0.200	0.032	< 0.001 *	0.036	0.041	0.379	-	-	-

Notes: * α < 0.05
